# Simulation, A Fundamental Component of Training to Treat Placenta Accreta Spectrum

**DOI:** 10.1055/s-0042-1760216

**Published:** 2022-12-29

**Authors:** Albaro José Nieto-Calvache, Lorgio Rudy Aguilera

**Affiliations:** 1 Clínica de Acretismo Placentario, Hospital Universitario Fundación Valle del Lili, Cali, Colombia; 2 Departamento de Ginocología y Obstetricia, Hospital De La Mujer Dr. Percy Boland, Santa Cruz de la Sierra, Bolivia

Dear Editor,

We thank Professor Chikazawa et al. 1 for their interest in our paper 2 and for highlighting the importance of simulation during training for the management of placenta accreta spectrum (PAS). There are multiple options to manage PAS and although the disease exhibits a wide variety of clinical presentations (spectrum), most groups choose a single therapeutic alternative and apply it to all their patients, making it difficult to respond when deviations from the original plan arise. Few publications propose a clear sequence of interventions applicable to all types of PAS. Our group uses the protocolized approach described by Palacios-Jaraquemada et al. 3 applicable to patients with suspected prenatal PAS, but also to those diagnosed intraoperatively, considering the nature (predominantly hypervascularization or presence of vesicouterine fibrosis) and the topography of the lesion (which uterine wall is affected, and which is the relationship of the lesion with the vesicouterine peritoneal fold). 3 4 This protocol includes four steps ( Fig. 1 ).

**Fig. 1:**
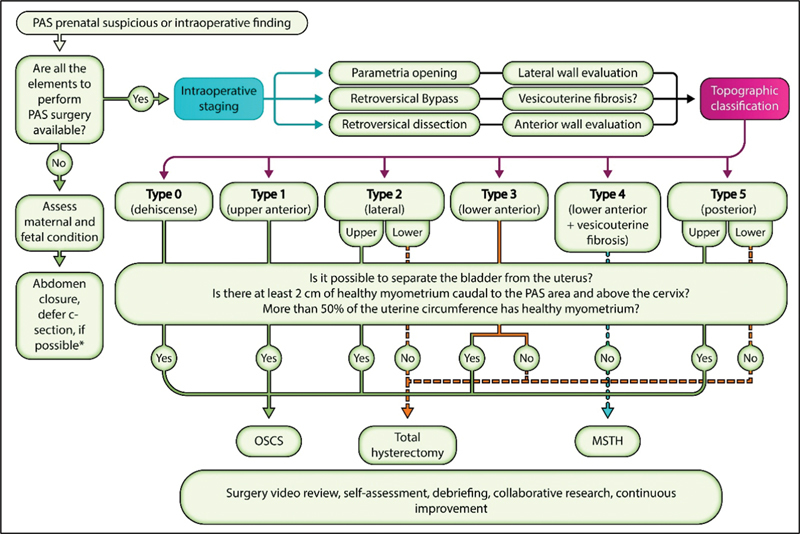
Protocolized approach to PAS. Abbreviations: MSTH, Modified subtotal hysterectomy; OSCS, One-step conservative surgery; PAS, Placenta accreta spectrum. *If the clinical condition of the patient or her fetus does not allow to defer the procedure, avoid manipulating the placenta.

First, the evaluation of the available resources and the clinical situation of the patient (to define whether or not to go ahead with the surgery). Doctor Chikazawa et al. 1 rightly point out that the process of training to manage PAS is a long one, and that obstetricians without such training are likely to be faced with the intraoperative finding of PAS. As useful as training in what to do, it is necessary to be very clear about what to avoid in the event of a PAS intraoperative finding, without the appropriate resources (human or technological), the greatest success of the obstetrician would be to avoid a high number of interventions when the clinical situation of the patient allows it.

Second, intraoperative staging through 4 actions: opening of the parametrium (to evaluate the lateral uterine wall), digital evaluation of the retrovesical space (Pelosi maneuver), dissection of the retrovesical space by ligating the vesicouterine pedicles (to evaluate the anterior uterine wall), and exteriorization of the uterus to evaluate the posterior uterine wall.

Third, the recommended treatment will be chosen (one step conservative surgery, total hysterectomy or modified subtotal hysterectomy) based on the topographic classification, 3 4 and after answering the three following questions: Is it possible to separate the bladder from the uterus? Is there > 2 cm of healthy myometrium cephalic to the cervix and caudal to the PAS area? Does > 50% of the circumference of the uterus (in an axial section at the level of the PAS area) has healthy myometrium? ( Fig. 1 ).

Fourth and last, it is essential to have photographic and video recording elements of the surgical procedures to later on debrief, self-assess, and provide research activities that facilitate learning and continuous improvement of the performance of the group. A standardized approach facilitates the construction of a mental map that obstetricians can internalize or consult immediately, facilitating decision-making in the face of a planned or unexpected PAS case.

## References

[1] Chikazawa K Matsubara S Kuwata T Difficulties in the management of placenta accreta spectrum disorders are not confined to low-/middle-income countries: a possible usefulness of simulation training Rev Bras Ginecol Obstet 2022 44 08 804 805 10.1055/s-0042-1751073 36075226 PMC9948059

[2] Aguilera L R Mojica-Palacios L M Urquizu F Gorena M Guzmán F T Galliadi L MV Difficulties in the management of placenta accreta spectrum in hospitals with limited resources Rev Bras Ginecol Obstet 2022 44 05 467 474 10.1055/s-0042-1742408 35472821 PMC9948092

[3] Palacios-Jaraquemada J M Fiorillo A Hamer J Martínez M Bruno C Placenta accreta spectrum: a hysterectomy can be prevented in almost 80% of cases using a resective-reconstructive technique J Matern Fetal Neonatal Med 2022 35 02 275 282 10.1080/14767058.2020.1716715 31984808

[4] Nieto-Calvache A J Palacios-Jaraquemada J M Aryananda R A Rodriguez F Ordoñez C A Bryon A M How to identify patients who require aortic vascular control in placenta accreta spectrum disorders? Am J Obstet Gynecol MFM 2022 4 01 100498 10.1016/j.ajogmf.2021.100498 34610485

